# A partially supervised physical activity program for adult and adolescent survivors of childhood cancer (SURfit): study design of a randomized controlled trial [NCT02730767]

**DOI:** 10.1186/s12885-017-3801-8

**Published:** 2017-12-05

**Authors:** Corina S. Rueegg, Susi Kriemler, Simeon J. Zuercher, Christina Schindera, Andrea Renner, Helge Hebestreit, Christian Meier, Prisca Eser, Nicolas X. von der Weid

**Affiliations:** 1 Oslo Centre for Biostatistics and Epidemiology, Oslo University Hospital and Institute of Basic Medical Sciences, University of Oslo, Sognsvannsveien 9, 0372 Oslo, Norway; 2 Department of Health Sciences and Health Policy, University of Lucerne, Frohburgstrasse 3, 6002 Lucerne, Switzerland; 3 Epidemiology, Biostatistics and Prevention Institute, University of Zürich, Hirschengraben 84, 8001 Zürich, Switzerland; 4 Department of Pediatric Oncology and Hematology, University Children’s Hospital Basel (UKBB), University of Basel, Spitalstrasse 33, 4056 Basel, Switzerland; 5 Institute of Social and Preventive Medicine, University of Bern, Finkenhubelweg 11, 3012 Bern, Switzerland; 6 Paediatric Endocrinology, Pediatric Endocrinology Centre Zurich AG (PEZZ), Möhrlistrasse 69, 8006 Zürich, Switzerland; 7 Children’s Hospital, University Hospital Würzburg, Josef-Schneider-Str. 2, 97080 Würzburg, Germany; 8 Division of Endocrinology, Diabetes, Metabolism and Bone Research, University Hospital Basel, Missionsstrasse 24, 4055 Basel, Switzerland; 9 University Clinic of Cardiology, Preventive Cardiology and Sports Medicine, Inselspital, University Hospital Bern, 3010 Bern, Switzerland

**Keywords:** Randomized controlled trial, Physical activity, Exercise intervention, Childhood cancer survivors, Late-effects, Cardiovascular disease, Bone health, Body composition, Physical fitness, Quality of life

## Abstract

**Background:**

Beyond survival of nowadays >80%, modern childhood cancer treatment strives to preserve long-term health and quality of life. However, the majority of today’s survivors suffer from short- and long-term adverse effects such as cardiovascular and pulmonary diseases, obesity, osteoporosis, fatigue, depression, and reduced physical fitness and quality of life. Regular exercise can play a major role to mitigate or prevent such late-effects. Despite this, there are no data on the effects of regular exercise in childhood cancer survivors from randomized controlled trials (RCTs). *Primary outcome* of the current RCT is therefore the effect of a 12-months exercise program on a composite cardiovascular disease risk score in childhood cancer survivors. *Secondary outcomes* are single cardiovascular disease risk factors, glycaemic control, bone health, body composition, physical fitness, physical activity, quality of life, mental health, fatigue and adverse events (safety).

**Methods:**

A total of 150 childhood cancer survivors aged ≥16 years and diagnosed ≥5 years prior to the study are recruited from Swiss paediatric oncology clinics. Following the baseline assessments patients are randomized 1:1 into an intervention and control group. Thereafter, they are seen at month 3, 6 and 12 for follow-up assessments. The intervention group is asked to add ≥2.5 h of intense physical activity/week, including 30 min of strength building and 2 h of aerobic exercises. In addition, they are told to reduce screen time by 25%. Regular consulting by physiotherapists, individual web-based activity diaries, and pedometer devices are used as motivational tools for the intervention group. The control group is asked to keep their physical activity levels constant.

**Discussion:**

The results of this study will show whether a partially supervised exercise intervention can improve cardiovascular disease risk factors, bone health, body composition, physical activity and fitness, fatigue, mental health and quality of life in childhood cancer survivors. If the program will be effective, all relevant information of the SURfit physical activity intervention will be made available to interested clinics that treat and follow-up childhood cancer patients to promote exercise in their patients.

**Trial registration:**

Prospectively registered in clinicaltrials.gov [NCT02730767], registration date: 10.12.2015.

**Electronic supplementary material:**

The online version of this article (10.1186/s12885-017-3801-8) contains supplementary material, which is available to authorized users.

## Background

Thanks to improvements in diagnosis, treatment and supportive care of childhood cancer patients, 5-year survival rates have increased drastically since the 1960s and reached 81% in the last decade in Europe [1, 2], including Switzerland [3]. However, childhood cancer survivors (CCS) are at risk to develop a series of physical or psychological late-effects, either directly as a result of the tumour and the aggressive treatments received, or secondary due to an unfavourable lifestyle [4–6]. The authors of a recent study estimated that 96% of CCS suffer from any chronic health condition and 81% from a serious or life-threatening chronic disease by the age of 45 years [7]. These late-effects shift the focus of modern childhood cancer treatment and research from pure survival to long-term functionality, health and quality of life [8].

Based on evidence from the general population or adult cancer survivors, we can hypothesise that regular physical activity has the potential to decrease the survivors’ risk for late-effects, such as cardiovascular diseases (CVD) [9–11], stroke [11, 12], second cancers [11, 13], obesity [11, 14], dyslipidaemia [15, 16], insulin resistance and diabetes mellitus [11, 17], osteoporosis [18–21], depression [22, 23], and cognitive decline [24, 25]. Despite these encouraging findings on benefits of exercise in various populations, specific studies on physical activity interventions in adult or adolescent survivors of childhood cancer aiming to reduce late-effects and increase physical activity are scarce. Small controlled exercise interventions over 2–4 months have shown beneficial effects on fatigue [26], metabolic risk factors and fitness [27], but to date no randomized controlled studies have been published.

Furthermore, traditional physical activity interventions in any field usually focus on specific types of exercises (such as strength training, tai chi, etc.) and participants are invited several times per week to join exercise sessions [28]. The problem of such a supervised and standardized approach is that the increase in physical activity is often not maintained by individuals after the intervention has ended and does therefore not lead to a sustained change in their behaviour [28, 29]. Our study is novel in applying an individual and motivational feedback-based approach with a personalized exercise counselling and program embedded in each participant’s daily life. Such an intervention may have a higher potential to result in a lasting behaviour change towards an active lifestyle and therefore ameliorate physical and psychological late-effects.

## Methods/design

This study protocol is written in accordance with the SPIRIT guidelines [30] (see the SPIRIT Checklist in Additional file 1).

### Study objectives

The primary objective of the proposed study is to evaluate the effect of a partially supervised and personalized physical activity program on the cardiovascular disease (CVD) risk of adolescent and adult survivors of childhood cancer in a randomized controlled trial. Secondary objectives are to assess the effect of the physical activity program on single CVD risk factors, glycaemic control, bone health, body composition, physical fitness, physical activity, quality of life, mental health, fatigue and adverse events (safety).

### Primary outcome

The primary outcome of the randomized controlled trial (RCT) is defined as the change in a composite CVD risk score [31, 32] from baseline to 12 months in the participants of the intervention group compared to the participants of the control group. The composite score is based on the assumption that a physical activity intervention shall have overall beneficial effects on the cardio-metabolic risk, affecting most if not all components of the metabolic syndrome. We chose a composite CVD risk score because the prevalence of single components of the metabolic syndrome is low in adolescents and young adults. A longitudinal study showed that a clustered score in adolescents predicted metabolic syndrome in adulthood [33] and was sensitive to change by a physical activity intervention in youth [34]. The composite CVD risk score will be calculated by averaging the z-scores based on gender- and age-specific external references of all components of the metabolic syndrome, including waist circumference, blood pressure, homeostatic model assessment insulin resistance [HOMA-IR], inverted high density lipoprotein cholesterol, triglycerides and cardiorespiratory fitness [32, 34–39].

### Secondary outcomes

Secondary outcomes are differences in change between the intervention and control group from baseline to 6 months for the composite cardiovascular disease risk score, and from baseline to 6 and 12 months for the single CVD risk factors, glycaemic control, bone health, body composition, physical fitness, physical activity, quality of life, mental health, fatigue and adverse events (safety). Additional file 2: Table S1 lists all assessed outcome variables.

### Study design

**Fig. 1:**
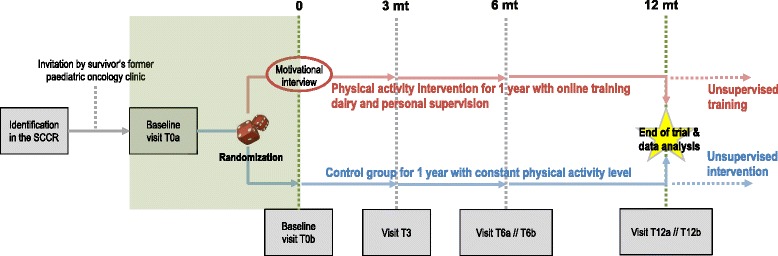
SURfit study design. Shows the general design and procedure of the SURfit study. All visits of T0, T3, T6 and T12 are at the University Children’s Hospital Basel (UKBB) including a visit at the Bone Research Unit of the University Hospital Basel (USB) to perform the DXA and pQCT scans (T0a and T12a). After one year of trial, participants of the control intervention who wish to, can receive the same personalized exercise counselling with motivational tools but no personal follow-up coaching. Participants of the intervention group will hopefully continue their training without supervision of the study team but still having access to the motivational tools of the study. Abbreviations*:* DXA, dual x-ray absorptiometry; mt, months; oGTT, oral glucose tolerance test; pQCT, peripheral quantitative computed tomography; SCCR, Swiss Childhood Cancer Registry; T0a, initial baseline visit; T0b, second visit for baseline assessments; T3, assessment after 3 months; T6a and T6b, first and second visit of assessments after 6 months; T12a and T12b, first and second visit of assessments after 12 months

This study is a single-centre RCT including childhood cancer survivors from various paediatric oncology clinics of Switzerland. Control and intervention arms run parallel (Fig. 1). Assessments are performed at baseline (T0) and after three (T3), six (T6), and 12 (T12) months. The assessments at T0, T6 and T12 comprise of two visits (a and b, respectively) in the study centre, 14 days apart, and T3 of one visit. Randomization is performed after the first visit of T0 (T0a) (Additional file 2: Table S1**,** Fig. 1). A motivational interview for the intervention group is performed at the second visit of T0 (T0b). After the intervention period, controls are offered a similar personalized exercise programme without long-term coaching (see paragraph on physical activity intervention). Eligible participants are contacted, informed and, if consenting, enrolled into the study (see paragraph on recruitment) until we reach the pre-defined number of participants.

### Ethics

The study was approved by the Swiss Ethics Committee on research involving humans (Ethikkommision Nordwest- und Zentralschweiz [EKNZ]). Informed Consent as documented by signature is obtained from each survivor prior to participation in the study. Data protection is assured by pseudonymization and can only be decrypted by study personnel involved in this research.

### Study population / inclusion criteria

Eligible participants are identified by the Swiss Childhood Cancer Registry (SCCR) [40, 41]. SURfit includes CCS aged <16 years at diagnosis, diagnosed with a cancer classifiable according to the International Classification of Childhood Cancer (ICCC-3) [42] or Langerhans Cell Histiocytosis, who were diagnosed and treated at a clinic of the Swiss Paediatric Oncology Group (SPOG), survived ≥5 years since primary cancer diagnosis or any subsequent cancer event (relapse or 2nd tumour), and, are aged ≥16 years at baseline (T0a) of the study. Participants have to agree that they will commit to the conditions of their study group allocation prior to the allocation and independent of the allocation.

The presence of any of the following criteria, assessed at baseline (T0a), leads to exclusion of the participant: participation in another clinical trial, inability to exercise or exercise potentially harmful, pregnant or breast feeding, cardiac arrhythmias under exercise, diagnosis of diabetes <3 months previously, detection or presence of a clinical condition that needs immediate treatment, planned surgeries within the subsequent 12 months that interfere with physical exercising, major musculoskeletal injuries/fractures <2 months previously, change in medication that interfere with the parameters of the CVD risk score < 1 month previously, >4 h of reported vigorous physical activities per week, or, inability to follow the procedures and understand the intervention and assessments of the study, e.g. due to cognitive impairment, language problems, or psychological disorders.

### Recruitment

Eligible patients are contacted with an information letter from their former treating hospital including a short study information brochure. Interested survivors then receive the detailed patient information of the study and the informed consent. Survivors decide upon this information whether or not they want to participate in the study. A study hotline or the responsible investigator can be contacted to clarify remaining questions about the study. All survivors who do not react to the study invitation are followed-up by a phone call and asked about their interest in the study. Survivors who decide to participate are invited for the baseline assessment where final decision upon eligibility is made and the informed consent is obtained.

We record the reasons for non-participation of each contacted survivor who does not want to participate. In addition, basic information on demographics and clinical factors are available from the SCCR on non-participants. This will allow us to get information on the representativeness of the participants in the study.

### Randomization

To ensure high quality randomization and even distribution of important prognostic factors among the intervention and the control group, a web-based minimization randomization approach (Randomizer: Institute for Medical Informatics, Statistics and Documentation, Medical University of Graz, Austria; available at www.randomizer.at) is used for the 1:1 allocation of participants into the two study arms [43]. We include gender and four categories according to the initial cancer diagnosis (leukaemia and lymphoma; CNS tumours; bone tumours and soft tissue sarcomas; other diagnoses) as grouping variables in the randomization. An external collaborator who is independent of the patient recruitment and enrolment process runs the randomization and treatment allocation (Additional file 2: Table S1).

### Measurements

Additional file 2: Table S1 gives an overview of the measurements and procedures at each time point of the study; Additional file 3 summarizes the measurements and respective methods used. Standard operating procedures (SOP) were developed for each measurement prior to the beginning of the study to reach a high level of standardisation and reliability. All assessors were extensively trained for the specific methods including pilot assessments prior to the study. Regular internal inspections of the assessments are carried out to maintain high methodological quality.

Study data are collected and managed using REDCap electronic data capture tools hosted at the Clinical Trials Unit, University of Bern [44]. All self-reported questionnaires are filled in by the participants directly in the REDCap database with a personal login during the study visits. All data entries are double-checked for consistency, errors and completeness by a data monitor. The data monitor, a member of the study team but independent of all the assessments, notifies any problem encountered back to the assessor using the specified feature in REDCap.

### Bloods

Fasted blood samples are taken in the morning after an overnight fast of at least 8 h. Glucose and glycated haemoglobin (HbA1c) are analysed within a few hours after sampling at the laboratory of the University Hospital of Basel. The other parameters assessed in the blood serum and plasma are centrifuged, divided into 1.0 ml aliquots and stored at −70 °C to be analysed at a later time point when a test kit can be completed at a the certified laboratory of the Endonet and Bone Research Unit, Basel.

### Cardiovascular disease risk

#### Blood pressure

Systolic and diastolic blood pressure is measured in sitting position on the left upper arm after at least 5 min rest using an automated oscillography (DINAMAP® ProCare [GE Medical Systems, Tampa, Florida, USA]). Based on the recommendations of the American Heart Association, the mean of two readings with a one-minute interval between them are recorded [45]. If the difference between the two readings is >5 mmHg, another two readings are taken [45].

#### Anthropometry

Standing height and weight are taken by standard procedures, barefoot and in underwear. Height is determined to the nearest 0.5 cm, weight is determined to the nearest 0.1 kg. Waist circumference is measured with a medical measuring tape to the nearest 0.5 cm. It is measured at the narrowest part of the torso (the middle between lower rib arch and spina iliaca) in relaxed, standing position at the end of expiration [46, 47]. Skinfold (SF) thickness is measured in triplicate to the nearest 0.2 mm with a Harpenden calliper (Harpenden Skinfold Calliper [Baty International, West Sussex, United Kingdom]) on the right body site at sites over triceps, biceps, subscapular and suprailiacal based on standard procedures [48, 49]. The sum of the four SF (each averages of the three readings) is taken to calculate absolute [kg] and relative [%] body fat by a formula validated against underwater weighing [49]. With this formula, body fat can be estimated with 3–5% error [49]. Lean body mass is derived from total body mass and body fat. Furthermore, percent fat mass, absolute fat mass and lean body mass (total body and regional) are estimated by whole body dual x-ray absorptiometry (DXA) using a Hologic Discovery densitometer (Hologic, Bedford MA, USA) [50, 51]. Body composition estimation from DXA scan shows good precision with a 2–3% coefficient of variation (CV) [52]. Muscle cross-sectional area (CSA) [cm^2^ and z-scores] at proximal tibia and radius is assessed using peripheral Quantitative Computed Tomography (pQCT, Stratec XCT 2000 scanner; Stratec Medical Pforzheim, Germany). Muscle CSA is obtained by subtracting fat CSA and bone CSAs from the CSA of the total limb. Precision error for muscle CSA determination of the calf by pQCT was found to be between 0.5%–4.1% [53–55].

#### Glycaemic control

Serum levels for insulin and C-peptide are determined by chemiluminescent enzyme immunoassays (ECLIA). The reproducibility based on the Elecsys 2010 Analyzer is 2.5–2.8% for insulin and 1.8–5.0% for C-peptide, respectively. Glucose is measured by the hexokinase method (Modular), consisting of a control unit, a core unit and an analytic ISE unit (E-module). Insulin resistance (IR) is estimated by calculating homeostasis model assessment (HOMA-IR) index (fasting serum insulin [μU/ml] × fasting plasma glucose [mmol/l)/22.5]) [56]. HbA1c is measured by a high efficiency fluid chromatography HPLC (VG8). In addition, 2 h 75 g oral glucose tolerance test (oGTT) after an overnight (≥8 h) fast is done to assess insulin resistance [57–59].

#### Blood lipids

Total cholesterol, high-density lipoprotein (HDL) cholesterol, low-density lipoprotein (LDL) cholesterol and triglycerides are measured by standard method on an auto-analyser (COBAS Integra 800; Roche Diagnostics, Basel, Switzerland). Intra-assay and inter-assay CVs are between 1.6–2.2% for total cholesterol, 2.4–3.6% for HDL, 0.7–2.1% for LDL, and 1.1–3.7% for triglycerides, respectively.

### Bone health

#### Bone mineral content and density

Bone mineral content (BMC, g), bone area (BA, cm^2^) and areal bone mineral density (aBMD, g/cm^2^) are measured at the lumbar spine, femoral neck and hip by DXA using a Hologic Discovery densitometer (Hologic, Bedford MA, USA) according to existing guidelines [60]. For aBMD evaluation at the lumbar spine, mean aBMD data of vertebral bodies L1 to L4 are reported unless vertebral bodies showing artefacts need to be excluded. A single densitometer is used throughout the study. In a previous study, the CV of individual measurements was 1.1% for the spine, 1.4% for the femoral neck, 1.9% for the trochanteric region, and 1.1% for the total hip [61]. The total radiation exposure of a DXA scan is 3–6 μSv.

#### Trabecular bone score (TBS)

Measurement is performed using spine DXA files with the TBS iNsight Software (version 1.8; Med-Imaps, Pessac). The software uses the antero-posterior spine raw image(s) from the densitometer, including the BMD region of interest and edge detection so that the TBS calculation is performed over exactly the same region of interest as the aBMD measurement. It assesses the heterogeneity of the areal density, with a higher heterogeneity implying poorer trabecular connectivity. Short-term reproducibility (CV) for TBS is reported to be 2.1% and 1.7% for spine aBMD in 92 individuals with repeat spine DXA scans performed within 28 days [62, 63].

#### Vertebral fracture assessment (VFA)

Is an established, low radiation method for detection of prevalent vertebral fractures using DXA [64]. Lateral spine scans are performed simultaneously with aBMD measurements using the Hologic Discovery densitometer. Spine fractures are classified using the standard semi quantitative scoring system of Genant and colleagues [65]. This scoring system differentiates three fracture grades based on the height reduction of the affected vertebral body (grade 1: 20–25%; grade 2: 25–40%, grade 3:>40%) [65].

#### Bone architecture & strength

Volumetric bone density (vBMD), bone mass, and bone geometry is measured using pQCT (Stratec XCT 2000 scanner; Stratec Medical Pforzheim, Germany) at the distal epiphysis and diaphysis of the non-dominant lower leg and lower arm. We will assess bone total CSA in mm^2^ at the epiphyseal and diaphyseal sites, cortical CSA (excluding the medullary CSA) in mm^2^ at the diaphyseal sites, total and trabecular vBMD in mg/cm^3^ at the epiphyseal sites, and cortical BMD in mg/cm^3^ at the diaphyseal sites [66, 67]. Absolute values are transformed in z-scores based on reference values [68, 69].

Quantitative computed tomography measures attenuation of x-rays projected through the limb at one-degree steps covering 180 degrees, resulting in an image of the cross-section of the limb. The slice thickness of 2 mm allows a three-dimensional (volumetric) assessment of bone density. X-ray attenuation is linearly transformed into hydroxyapatite (HA) densities. Unlike some other pQCT scanners, the Stratec XCT 2000 is calibrated with respect to water which is set at 60 mg HA, so that fat results in 0 mg HA [70]. HA equivalent densities are automatically calculated from the attenuation coefficients by employing the manufacturer’s phantom which itself is calibrated with respect to the European Forearm Phantom (EFP; QRM, Erlangen, Germany) [70]. The effective radiation dose is 0.2 μSv per scan and per scout view as indicated by the manufacturer.

Radius bone length is set equal to ulnar length, which is measured to the nearest 5 mm with a measuring tape by palpation of the olecranon and the ulnar styloid. Tibia length is measured from the medial knee joint cleft to the end of the medial malleolus. A scout view of the distal end of tibia and radius is performed and the automated detection algorithm provided by the manufacturer is used to place the reference line at the distal bone end. Two scans are performed for each of radius and tibia: one at 4% of total bone length measured from the reference line of the scout view in the distal epiphysis, and one at 66% of total bone length in the proximal part of the diaphysis. A reproducibility study based on 9 subjects with 4 repeated measurements defined the smallest detectable differences (1.96 × Standard Deviation [SD]) to be 4.74 and 3.92 mg/cm^3^ for trabecular vBMD at the radius and tibia, respectively, and 11.68 and 5.39 mg/cm^3^ for total vBMD at the radius and tibia, respectively [71].

#### Bone metabolism and hormones

Biomarkers of bone metabolism provide a quantitative measure of the relationship between bone deposition and resorption. Measuring the balance between deposition and resorption in relation is the basis of explaining change in BMD over time and can be taken as determinant of BMD change. The following biochemical markers of bone turnover are assessed: a) bone formation markers: bone-specific alkaline phosphatase (BAP), osteocalcin (OC), N-terminal propeptide of type I procollagen (PINP); b) bone resorption markers: C-terminal telopeptide of type I collagen (CTX). Serum BAP (IDS-iSYS Ostase BAP) as well as 25-hydroxy-vitamin D3 (IDS-iSYS 25-Hydroxy Vitamin D) will be determined using an enzyme-immunoassay (EIA) on the IDS-iSYS (Immunodiagnostic Systems, Frankfurt/Germany). The intra- and inter-assay variations are <9% for BAP [72, 73], and 3.6% and 16.9% for 25-hydroxy-vitamin D3 [74, 75], respectively. The parameters beta-CrossLaps (CTX), N-MID-Osteocalcin, PINP and intact parathyroid hormone (iPTH) are measured in serum with ECLIA on the automated analyser Elecsys 2010 (Roche Diagnostics, Rotkreuz, Switzerland) [76, 77]. The intra- and inter-assay variations are 2.4–7.2% for CTX, 1.1–5.9% for OC, 1.7–4.0% for PINP, and 1.7–5.5% for iPTH, respectively [61]. Uncarboxylated OC (ucOC) is measured after previous incubation of serum samples with hydroxyapatite (5 mg/ml) to separate out carboxylated OC (cOC) from the ucOC as previously described [78]. The ucOC in the supernatant is measured using the same assay as for total osteocalcin and will be reported as a concentration and as a fraction of the total [79].

Further hormone analyses including thyroid-stimulating hormone (TSH), free thyroxine (fT4), gonadotropins (luteinizing hormone [LH], follicle-stimulating hormone [FSH]), estradiol (E2), total testosterone (TT) and cortisol will be carried out using ECLIA on the automated analyser COBAS e411 (Roche Diagnostics, Rotkreuz, Switzerland). The intra- and inter-assay variations are 1.5–8.7% for TSH, 1.8–7.6% for fT4, 0.8–5.2% for LH, 1.8–5.3% for FSH, 2.4–11.9% for E2, 1.2–8.4% for TT, and 1.1–1.6% for cortisol, respectively [80].

Insulin like growth factor 1 (IGF-1) and insulin like growth factor binding protein 3 (IGF-BP3) are determined using ECLIA on the IDS-iSYS (Immunodiagnostic Systems, Frankfurt/Germany). The intra- and inter-assay variations are 6.0% and 9.8% for IGF-1, and 7.9% and 15.5% for IGF-BP3, respectively [81]. All assays are performed in duplicate by using the same biomarker kit, and the mean value will be recorded.

#### Nutrition

To correctly interpret bone health of the subjects, the relevant parameters like calcium intake [mg/day], protein intake [g/day] and vitamin D (supplement intake, sun exposure and nutrition) are also assessed with standardized and validated self-reported questionnaires [82, 83].

### Physical fitness

#### Aerobic fitness

The participants complete a continuous incremental cycling test to volitional exhaustion following the step protocol proposed by Godfrey and colleagues [84] in accordance to the international guidelines for exercise testing [85, 86]. Work rate is increased every minute by 20 W with an initial load of 20 W. At each visit, a 12-lead electrocardiogram (ECG, Schiller CS-200, Schiller AG, Baar, Switzerland) at rest is performed to rule out relevant arrhythmias and other pathologies that may pose a risk to the patient during the cycling test and/or intervention. All participants are tested using a calibrated cycle ergometer (Ergospirometrie-System CS-200 and SCHILLER ERG 911S Plus cycle ergometer [SCHILLER AG], Baar, Switzerland) and metabolic cart (LF8 PowerCube®-Ergo Gas-Analysator [Ganshorn Medizin Electronic], Niederlauer, Germany). The metabolic cart is calibrated before each exercise test with two gases of known concentrations. Peak oxygen uptake (VO_2_peak) is determined by the highest VO_2_ averaged over 30s during the test. Peak performance (Wpeak) is defined as the power maintained over the final 1-min stage of the test plus 5 W for each fulfilled 15 s bout of the non-finished stage. Maximal aerobic power and VO_2_peak will be expressed in percent of predicted [87]. Electrocardiography using chest leads and oxygen saturation measured at the finger (Masimo SET-Monitor Radical-7, [Masimo Corporation], Irvine, USA) are used to monitor the participant throughout the test for safety reasons and to determine maximal heart rate and desaturation under exercise. The test is terminated according to existing guidelines [86]. Blood pressure and Borg Rating of Perceived Exertion (RPE) are assessed at the end of each stage [88, 89]. In addition, heart rate, blood pressure and Borg RPE are assessed 1, 2, and 3 min post exercise to assess recovery as a marker of physical fitness [90].

#### Muscular strength

To assess muscular strength of the lower body, the 1-min sit-to-stand (STS) test is performed [91, 92]. The participants perform one test trial at least 20 min before the final test. The number of repetitions of standing up and sitting down from a chair in the final test is recorded. In addition, Borg RPE is assessed at the end of the test [88, 89]. The test is performed on a height adjustable chair to ensure a 90° knee angle. The 1-min STS test showed high reliability and good criterion related validity with other exercise capacity tests such as the 6-min walk test or stair climbing [93–96]. Existing population-based reference values from Switzerland will help to identify subjects with decreased lower body muscular strength and endurance [91].

Upper body strength is assessed through a handgrip strength test using the JAMAR Hydraulic Hand Dynamometer (Lafayette Instrument®, Lafayette, USA). The JAMAR dynamometer was validated in several studies and is regarded as the gold standard in measuring handgrip strength [97]. The dynamometer assesses force in kg (0–90 kg) to the nearest 2 kg. Hand grip strength is measured according to the American Society of Hand Therapists (ASHT) recommendations in a sitting position on a height adjustable chair with the dynamometer resting on a table in front of the subject [98]. Each hand is measured 3 times, with alternating sides, starting with the right hand and one minute breaks between measurements. A 1-min break is described as sufficient in the literature in order to yield consistent values [99].

### Physical activity

#### Pedometer

Participants wear a pedometer (Fitbug Air®, Fitbug, London, United Kingdom) between the two visits of T0, T6 and T12, to count number of daily steps (divided into overall steps and aerobic steps) [100]. The steps per day of the preceding 14 days are stored in the device and entered into the REDCap database when the participant returns the device at the second visit.

In addition, participants of the intervention group will wear a pedometer and record their daily steps throughout the entire study period (see paragraph on physical activity intervention).

#### Accelerometer

Physical activity (PA) is assessed by accelerometer (ActiGraph, Pensacola, Florida, USA) which will be worn on the right hip during at least 7 days between the two visits of T0, T6 and T12. The device measures accelerations of ±6 G. The sample rate of the accelerometer will be set to measure raw signals at 100 Hz. These are then translated into either metabolic energy equivalents of no, light, moderate and vigorous physical activities to estimate the effect on the metabolic outcomes or in cumulative impacts per day for the bone outcomes. Data are included if at least four full days (including at least one weekend day) with a minimum of 10 h are measured [101]. Participants are asked to wear the accelerometer also at night to capture sleeping time. Validity of accelerometer is good with correlation coefficients of 0.65 between accelerometer assessed metabolic energy equivalents and indirect calorimetry [102], and 0.74 between accelerometer impact loading and ground reaction forces by force plates [103]. For metabolic outcomes, overall PA will be expressed as total PA (total counts), average PA in counts/min, as well as time (min/d) sedentary and in light, moderate and vigorous PAs according to proposed, previously published cut-off levels [101, 102, 104]. For bone outcomes, impact loading will be expressed as cumulative impacts per day (n/day) >2, 3, 4, 5 and 6 G, respectively. Less than 100 impact loadings >3.9 G per day were shown to be effective to increase bone mineral density in premenopausal women with higher effects in those with low baseline values [105]. Such a level is reached with jogging, fast running and jumping activities.

#### Questionnaire

The Seven-Day Physical Activity Recall questionnaire (PAR), the Exercise Motivations Inventory (EMI-2) and a self-constructed questionnaire including items of the Lipid Research Clinics questionnaire (LRC) are used to assess the time individuals engage in physical activity, their reasons for exercising, and type and time of sports. The PAR assesses the time an individual engaged in moderate, hard, very hard activities and sleep during the 7 days prior to the assessment [106]. The PAR was validated in several studies against objective measures of physical activity and showed satisfying psychometric properties in different populations such as children and adults [106–109]. The EMI-2 is a validated scale to measure an individual’s reason for exercising. It comprises of 44 items reflecting 12 dimensions including stress management, weight management, recreation, social recognition, enjoyment, appearance, personal development, affiliation, ill-health avoidance, competition, fitness and health pressures [110, 111]. The self-constructed questionnaire includes items on type and time of current sports and questions from the validated LRC [112].

### Quality of life and mental health

#### Health related quality of life

Is assessed using the Short Form-36 (SF-36) [113, 114]. This instrument is validated and has been successfully used in samples of long-term CCS [115–119]. It consists of 36 questions that can be summarized into eight scales: physical functioning, role limitation due to physical health (role limitation physical), bodily pain, general health perception, energy & vitality, social functioning, role limitation due to emotional problems (role limitation emotional), and mental health. The eight scales can be further aggregated into a Physical Component Summary (PCS) and a Mental Component Summary (MCS) [114]. We will convert raw scores into T-scores (mean = 50, SD = 10, range 0–100) according to age- and sex-stratified norm data from a public use-file of the German Federal Survey (*N* = 6964) because no Swiss data of the SF-36 are available [113].

#### Fatigue and actual well-being

Is assessed with a Visual Analogue Scale (VAS) and the Checklist Individual Strength (CIS). A VAS for fatigue and well-being is designed to measure these characteristics, which are believed to range across a continuum of values and cannot easily be measured directly. Operationally, the VAS is a horizontal line, 100 mm in length, anchored by two word descriptors at each end. In our study, the descriptors will range from ‘not tired at all’ to ‘completely exhausted’ for fatigue and from ‘feeling absolutely miserable’ to ‘perfect well-being’ for well-being. The CIS is a validated 20-item questionnaire, that is designed to measure four aspects of fatigue that may have been experienced during the previous 2 weeks, i.e. severity of fatigue (8 items), concentration (5 items), motivation (4 items) and physical activity (3 items) [26, 120, 121]. Each item is scored on a 7-point Likert scale. The total score is the sum of the scores 1–7 in the 20 items (range 20–140). Norm scores are available for different patient groups and healthy people. The CIS has also been successfully used in long-term survivors of childhood cancer [26]. The CIS showed to have good internal consistency and validity across studies and could successfully discriminate between non-fatigued and fatigued groups and cut-off points for clinical levels of fatigue have been developed [26, 122, 123].

#### Mental health

Psychological distress will be assessed using the Brief Symptom Inventory (BSI) [124]. The BSI is a widely used and well-validated instrument to screen the following nine domains of distress: somatization, obsessive-compulsive tendencies, interpersonal sensitivity, depression, anxiety, aggression, phobic anxiety, paranoid ideation, and psychotic tendencies. Responses to all 53 items can be further summarized in the Global Severity Index (GSI). For each item, participants express how much they agree with a statement describing the previous 7 days on a 5-point Likert-scale ranging from 1 (not at all) to 5 (very much). Scores from all scales will be transformed to T-scores (mean = 50, SD = 10, range 0–100) according to the German norm population [125]. A T-score of ≥63 on any scale corresponds to the 90th percentile of the norm population and indicates a risk for being at significant psychological distress in this area (case rule) [125].

### Personal history and clinical examination

A thorough personal history, study of the medical record and clinical examination is performed in each participant, with special emphasis on the cancer history, signs and symptoms of cardio-metabolic, pulmonary or neurological diseases, health behaviour, medical doctor visits, hospitalizations, and medications [126–128]. Symptoms, medical doctor visits, hospitalizations and medications are updated after 6 and 12 months. Physical examination of the lungs, heart, abdomen, joints, extremities, ears, mouth, lymph nodes and a detailed neurological status are performed at baseline and after 6 and 12 months. Vital parameters including heart rate and blood pressure are taken at every visit. Tanner stadium is assessed once at baseline and only repeated after 6 and 12 months if the participant is not fully mature at baseline [129]. Socio-demographic characteristics are assessed by questionnaire at baseline, health behaviours at baseline and after 12 months, using standardized questions from the Swiss Health Survey and the Swiss Census [126–128].

### Adverse events and exercise related complications

Every adverse event including exercise related complications [130] whether or not causally related with the exercise training will be monitored based on standardized procedures and followed until resolved [131]. Each adverse event is recorded in the REDCap database and classified based on The Common Terminology Criteria for Adverse Events (CTCAE) [131]. Serious adverse events are reported to the sponsor and the responsible independent ethics committee. An independent safety auditor of the University Children’s Hospital Basel (UKBB) is monitoring patients’ safety throughout the study period.

Participants who show an elevated blood pressure or a pathological oral glucose tolerance test at baseline or during a study visits, can enter/stay in the study, but are referred to the family physician or a specialist to get the appropriate treatment. In case of diabetes, the participant will enter the study once a stable condition is reached but earliest after 3 months.

Participants at risk for cardiac late-effects due to cardio-toxic childhood cancer therapy (anthracyclines and/or chest radiation) can enter the study normally, but are recommended to get a cardiac assessment with an adult cardiologist according to recent recommendations [132].

#### Physical activity intervention (intervention group)

Survivors in the intervention group are asked to add at least 2.5 h of intense physical activities per week. These should include 30 min of strength building exercises and 2 h of aerobic exercises per week. Exercise bouts lasting 20 min or longer are counted towards the total weekly training time. This “dose” of physical activity is based on the international recommendations of healthy physical activities from the Centre of Disease Control and Prevention (CDC; www.cdc.gov) [133].

Based on the initial exercise test, general health status and participant’s preferences and motivation, subjects of the intervention group receive a counselling at the second visit of the baseline assessment (T0b; Fig. 1 **,** Additional file 2: Table S1). A standardized approach is used to assess survivors’ preferences with respect to physical activities, identify possible barriers and determine the individual motivation to start specific activities. Based on this assessment, individualized physical activities are defined and implemented into the participant’s daily life. Survivors of the intervention group are also motivated to incorporate activities of moderate intensities into daily life such as walking instead of driving or climbing stairs instead of taking the escalator. Participants are also advised to reduce inactive behaviour such as television viewing, computer games, etc. with the aim of reducing 25% of their actual media time. The motivational interview is performed by one of the project physiotherapists who have been trained prior to the study.

For motivational reasons each survivor of the intervention group is equipped with a step counter (pedometer, Model Fitbug Air) and asked to document daily steps. Participants keep a daily training log using a web-based platform with individual anonymous logins. Data on 1) strenuous exercise performed that day (type of exercise, duration), 2) step counts (overall and aerobic steps), 3) media-related sedentary time and sleeping hours, and 4) mood and well-being are entered and graphically displayed to give the participants an immediate feedback about their progress. The participants receive a “reminder” message on his or her mobile phone or via email if no entries are made for three consecutive days. If there are no entries for a whole week, the survivor is contacted by phone by his “personal coach” (physiotherapist). There are also scheduled phone contacts after 1, 2, 4, 5, 8 and 10 months of the intervention to discuss compliance, motivation, and progress and to re-counsel the survivors on their training plan. Training logs and physical activity behaviour are checked and discussed during the clinic visits (at months 3 and 6), and exercise counselling is repeated.

After the 1-year assessment, participants of the intervention group can keep their step counters and will still have access to the web-based training log to report and view their activity data, but no further support is given from the research team. A follow-up after 1–2 years off trial is planned but this is not subject of the current study protocol.

#### Control group

The control population of this study is asked to keep its activity level constant over the one-year study period. With this study, we will be able to test the effect of *additional* physical activity compared to a “normal” activity level. After the one-year study period, participants of the control population have the opportunity to receive the same personalized physical activity counselling and motivational interview, but without personal follow-up coaching. They also receive a step counter and access to the same web-diary, which they can use to follow their physical activity plan.

#### Compliance

Compliance of the participants is assessed by different means. This will allow us to validate whether the aims of the intervention and control arm have been maintained and to make dose-response analyses for physical activity in this population.

Participants of the intervention group daily report the pedometer steps and sports performed in the web-diary, they are contacted immediately in case of non-compliance, they have monthly telephone contacts with the physiotherapists, and three-to-six-monthly assessment visits with the physicians, physiotherapists and other staff. After the one-year intervention, a structured interview is performed with the participants of the intervention group to assess their opinion on the intervention, their compliance, reasons for compliance or non-compliance and readiness to continue the physical activity programme. Furthermore, at each study visit (3, 6, and 12 months), the retained steps of the pedometers over 2 weeks prior to the visits are (unknown to the participants) downloaded and entered into the study database to assess how accurate participants of the intervention group report their daily steps in the web-diary.

Participants of the intervention and control group report their actual activities at each visit (T3, T6, and T12) and we objectively measure the activity levels at T6 and T12.

#### Blinding

With our physical activity intervention, it will not be possible to blind the study participants themselves, the project physiotherapists, the project physicians and some of the assessors. But wherever possible all other members of the project team will be blinded for group allocation of the participants, i.e. those who perform the DXA measurement, the physical performance test, the blood analysis, the quality check of the data in the database and the statistical analysis.

#### Sample size

A study by Kriemler and colleagues using the same CVD risk score showed a reduction in the z-score by 14% after a 1-year physical activity intervention in children and adolescents [34]. Our population is older with a history of cancer, intensive treatments and long hospitalisations. We therefore expect higher CVD risk in this population at baseline and even greater changes after an intervention. To be conservative, this study is powered to detect a difference between the intervention and control group of 15% (no change in the control group and 15% change in the intervention group) after the 1-year intervention. For a power of 0.80 and a two-sided type 1 error probability of 0.05, 60 survivors with complete data are required in each study arm. Assuming that 20% of participants will dropout or have missing data, 75 survivors have to be recruited in each arm. From the Swiss Childhood Cancer Registry we identified 4241 eligible 5-year survivors of whom about 1500 were diagnosed and treated in one of the three Swiss Paediatric Oncology Group (SPOG) clinics of initial recruitment (Basel, Lucerne, and Zurich) [3]. Therefore, with an expected participation rate of 20% of the survivors contacted and invited, we will reach a sufficient sample size.

#### Data analysis

Descriptive statistics will be used for clinical, sociodemographic and prognostic variables measured at baseline (stratified by intervention and control group). We will use frequencies and proportions with 95% confidence intervals (CI) for categorical variables and mean with ±SD or 95% CI (or median and range) for continuous variables. Descriptive analysis of baseline characteristics will be performed as soon as the baseline assessments are completed for all participants. This cross-sectional analysis will inform about the comparability of the treatment groups and the need for adjustment of between treatment group comparisons. Furthermore, baseline assessments will provide important results about characteristics, health behaviours and health status of long-term CCS in Switzerland. We will also compare the distribution of baseline sociodemographic characteristics to the Swiss Childhood Cancer Registry, to estimate the representativeness of our sample.

The primary analysis will be conducted as intention-to-treat analysis (ITT) for the primary outcome (change in composite CVD score from T0 to T12). All participants will be analysed in the group where they were originally allocated and missing data will be imputed by means of last observation carried forward (LOCF) [134]. LOCF is an appropriate procedure in this setting because it will lead to more conservative effect estimates (i.e. towards the null), because lost to follow-up is more likely to occur in the intervention group (because of an intensive and time-consuming programme) which is the group expected to change (improve). Attempt will be done to follow up all randomised participants, even if they withdraw from the allocated treatment. If the parameters of the composite CVD risk score are skewed, they will be transformed to reach normal distribution for calculating the z-scores.

The secondary analyses include several steps: *First*, we will do the same ITT analysis with appropriate means of imputation of missing data for all the secondary outcomes. *Second*, we will perform sensitivity analysis on the primary and all secondary outcomes, including, a) complete data (complete case analysis), i.e. observations with missing information on relevant variables will be dismissed; b) analysis of intermediate effects after 3 and 6 months of intervention; c) dose-response analysis based on the actual physical activities (min of vigorous physical activities per week) or physical performance (VO_2_peak) during the period of interest, independent of the group allocation; and, d) per protocol analysis.

Per protocol analysis will include several analyses based on the actual “treatment” that the participants adhered to (independent of group allocation), first based on the reported compliance and second based on the assumed compliance. In the first analysis based on the *reported compliance*, the following participants will be analysed as being in the “intervention group”: participants randomized into the intervention group who reported in the web-diary to have reached at least 2/3 of the target physical activity (addition of 100 min of moderate to vigorous physical activities [MVPA]); and, participants who were randomized to the control group but reported in the physical activity questionnaires to have increased their physical activity level by more than 30 min of extra MVPA per week during the period of interest. The rest of the participants will be analysed as being a “control”. The second analysis of *reported compliance* will include only the participants sufficiently compliant with the protocol that they were allocated to. It will include those randomized into the intervention group who completed at least 2/3 of training volume (e.g. the addition of 100 min per week of vigorous PA) and compare them to those randomized into the control group with no more than 30 min of extra vigorous PA per week than at baseline during the period of interest.

The analysis on the *assumed compliance* will be done according to the analyses of the reported compliance but with participants being defined as compliant if they reach an increase of ≥5% in VO2peak and/or ≥10% in the ventilatory anaerobic threshold from baseline [135].

Model selection Depending on the type of endpoint, mixed linear, logistic, Cox or Poisson regression will be used. All models will include the variables used in the adaptive randomization (age and former cancer diagnosis) and we will test the model assumptions and model fit. The models might be further adjusted for baseline values and important prognostic factors such as sex, socio-economic status, health-behaviours, treatments received, or former treating hospital if we observe an unequal distribution of important confounders between the treatment and control group.

The analysis will be done with Stata Statistical Software version 14 or newer (StataCorp LP, College Station, Texas, USA) and R Statistics (R Core Team, Vienna, Austria, www.R-project.org). A *p*-value <0.05 will be considered as statistically significant. Primary and secondary analyses will be performed when the data collection is finished for all participants.

#### Publication policy

Because of the large variety of outcomes assessed, we intend to publish the effect of the intervention on the different outcomes in more than one paper. We intend to publish the effect of the intervention on our primary outcome (CVD risk score) and related single cardiovascular disease risk factors, glycaemic control and body composition in a single paper (main paper). This publication will also include the changes in physical fitness and physical activity as well as the clinical status and safety endpoints. Beside this, we intend to publish the effect of the intervention in different papers of the following topics:

- Effect on bone health

- Effect on quality of life, mental health and well being

Results of the baseline assessments only and further in depth research questions will be published in additional papers of meaningful topics.

## Discussion

It’s estimated that 1 in 800 young adults under the age of 35 years living in developed countries is a survivor of paediatric cancer [2]. Unfortunately, studies have shown that the vast majority of these survivors present with chronic medical conditions such as cardiovascular and pulmonary diseases, 2nd cancers, overweight, osteoporosis, or psychological distress, directly impacting their late mortality, morbidity and quality of life [4, 7, 117, 136].

Physical activity and exercise have become a cornerstone in the prevention and treatment of chronic diseases including cancer, cardiovascular morbidity and mortality, diabetes, osteoporosis, fatigue or increasing psychological well-being [11, 16, 137–147] Even in palliative care, physical activity seems to reduce the burden of cancer- or therapy-related side effects [148]. Despite these striking evidences, there is a lack of randomized, controlled exercise trials aiming to reduce cancer- and therapy-related sequelae in paediatric cancer survivors, which may ultimately increase life expectancy, decrease morbidity and lead to an improvement in quality of life. This is especially important for childhood cancer survivors as these patients are young with a long life in front of them.

Besides contributing to the body of scientific knowledge, this randomized controlled trial will also use a novel approach with a personalized and partially supervised exercise intervention. The intervention is supported by multiple tools such as web-based feedback, pedometers and individually tailored counselling. With this approach, we try to overcome barriers and low compliance that is a major problem in the sustainability of intervention studies. Moreover, the measurement of most major cardio-metabolic risk factors and bone health and the inclusion of patient important outcomes like quality of life and psychological distress provide a complete picture.

Another new aspect is the precise measurement of bone architecture including a broad battery of possible determinants of osteoporosis or decreased bone mass in CCS. This may shed more light on the single and combined risk factors of increased bone fracture risk or osteoporosis in this population and on the potential preventive approaches.

The planned physical activity intervention has the potential to become an important pillar in the support of the whole CCS population. The project will evaluate whether a 1-year partially supervised program including exercise counselling with motivational feedback in CCS (aged 16 years and above) is able to reduce long-term sequelae and/or lifestyle-related diseases including but not limited to cardiovascular and metabolic risks as well as bone health, physical fitness, mental health, and quality of life. If the program shall prove effective, the SURfit material will become available to interested national and international clinics that treat and/or follow-up childhood cancer patients to promote exercise in their CCS.

## Additional files

Additional file 1: SPIRIT 2013 Checklist: Recommended items to address in a clinical trial protocol and related documents. Contains the SPIRIT Checklist in its original template. The table lists all the recommended items that should be addressed in a clinical trial study protocol and indicates the page numbers where the respective item is described. (DOCX 65 kb)

Additional file 2: Table S1. Schedule of enrolment, interventions and assessments within SURfit (DOCX 138 kb)

Additional file 3: Measures and methods within SURfit at different time points. The table in Additional file 3 describes in detail the methods used for each measurement in SURfit. It is an extension of the (Additional file 2: Table S1) in the paper. (DOCX 81 kb)

## Abbreviations

ASHT: American Society of Hand Therapists
BAP: Bone-specific Alkaline Phosphatase
BMC: Bone Mineral Content
BMD: Bone Mineral Density
BSI: Brief Symptom Inventory
CCS: Childhood Cancer Survivor
CDC: Centre of Disease Control and Prevention
CI: Confidence Interval
CIS: Checklist Individual Strength
CNS: Central Nervous System
cOC: Carboxylated Osteocalcin
CSA: Cross-sectional Area
CTCAE: Common Terminology Criteria for Adverse Events
CTX: C-terminal Telopeptide of Type I Collagen
CV: Coefficient of Variation
CVD: Cardiovascular Disease
DXA: Dual X-Ray Absorptiometry
E2: Estradiol
ECG: Electrocardiogram
EFP: European Forearm Phantom
EIA: Enzyme-Immunoassay
EKNZ: Ehtikkommision Nordwest und Zentralschweiz
EMI-2: Exercise Motivations Inventory
FSH: Follicle-stimulating Hormone
fT4: free Thyroxine
GSI: Global Severity Index
HA: Hydroxyapatite
HbA1c: Glycated Haemoglobin
HDL: High Density Lipoprotein Cholesterol
HOMA-IR: Homeostasis Model Assessment – Insulin Resistance
ICCC-3: International Classification of Childhood Cancer – 3rd Edition
IGF-1: Insulin Like Growth Factor 1
IGF-BP3: Insulin Like Growth Factor Binding Protein 3
iPTH: Intact Parathyroid Hormone
IR: Insulin Resistance
ITT: Intention to Treat Analysis
LDL: Low Density Lipoprotein Cholesterol
LH: Luteinizing Hormone
LOCF: Last Observation Carried Forward
LRC: Lipid Research Clinics questionnaire
MCS: Mental Component Summary
OC: Osteocalcin
oGTT: oral Glucose Tolerance Test
PA: Physical Activity
PAR: Seven-Day Physical Activity Recall Questionnaire
PCS: Physical Component Summary
PINP: N-terminal Propeptide of Type I Procollagen
p-QCT: Peripheral Quantitative Computed Tomography
RCT: Randomized Controlled Trial
RPE: Rating of Perceived Exertion
SD: Standard Deviation
SF: Skinfold
SF-36: Short Form-36
SOP: Standard Operational Procedure
SPOG: Swiss Paediatric Oncology Group
STS: 1-min sit-to-stand
SURfit: Survivor Fitness Study
T0, T3, T6, T12: Time point 0, Time point 4 months, Time point 6 months, Time point 12 months
TBS: Trabecular Bone Score
TSH: Thyroid-stimulating Hormone
TT: Total Testosterone
ucOC: Uncarboxylated Osteocalcin
UKBB: University Children’s Hospital Basel
VAS: Visual Analogue Scale
VFA: Vertebral Fracture Assessment
VO_2_peak: Peak Oxygen Uptake
Wpeak: Peak Performance in Watt
