# Supplementary material for: A partially supervised physical activity program for adult and adolescent survivors of childhood cancer (SURfit): study design of a randomized controlled trial [NCT02730767]
Source: BMC Cancer. 2017 Dec 5;17:822. doi: 10.1186/s12885-017-3801-8 (PMC5717834; doi:10.1186/s12885-017-3801-8)
Supplement: Supplementary file 2 — Schedule of enrolment, interventions and assessments within SURfit (DOCX 138 kb) [file 12885_2017_3801_MOESM2_ESM.docx]

**Table 1: Schedule of enrolment, interventions and assessments within SURfit**

|  | | **STUDY PERIOD** | | | | | |
| --- | --- | --- | --- | --- | --- | --- | --- |
|  | | Enrolment | Allocation | Post-allocation | | | Close-out |
| **TIMEPOINT** | | - 2 weeks (T0a) | 0 | 0  (T0b) | 3 mt  (T3) | 6 mt  (T6a/b) | 12 mt  (T12a/b) |
| **ENROLMENT:** | |  |  |  | | |  |
|  | Eligibility screen | x |  |  |  |  |  |
|  | Informed consent | x |  |  |  |  |  |
|  | Allocation |  | x |  |  |  |  |
| **INTERVENTIONS:** | |  |  |  | | |  |
|  | Physical activity motivational interview^a^ |  |  | x |  |  |  |
|  | Physical activity & exercise intervention^a^ |  |  |  |  |  |  |
|  | Control group |  |  |  |  |  |  |
| **ASSESSMENTS:** | |  |  |  | | |  |
| **Blood pressure / ECG** | |  |  |  | | |  |
|  | Systolic & diastolic blood pressure^b^ [mmHg] | x |  |  | x | x | x |
|  | 12-lead electrocardiogram [ECG] | x |  |  |  | x | x |
| **Anthropometry** | |  |  |  | | |  |
|  | Height [cm] | x |  |  | x | x | x |
|  | Body mass [kg] | x |  |  | x | x | x |
|  | Body Mass Index [z-score] | x |  |  | x | x | x |
|  | Waist circumference^a^ [cm] | x |  |  | x | x | x |
|  | Absolute [kg] and relative [%] body lean and fat mass by skinfold measurement | x |  |  | x | x | x |
|  | Absolute [kg] and relative [%] body fat mass by DXA measurement | x |  |  |  |  | x |
|  | Muscle cross-sectional area [cm^2^ and z-scores] at radius and tibia by pQCT | x |  |  |  |  | x |
| **Glycaemic control** | |  |  |  | | |  |
|  | Fasting insulin [mIU/l] |  |  | x |  | x | x |
|  | Fasting glucose [mmol/l] |  |  | x |  | x | x |
|  | Homeostasis Model Assessment Insulin Resistance (HOMA-IR)^b^ |  |  | x |  | x | x |
|  | Glycated haemoglobin (HbA1C) [mmol/mol] |  |  | x |  | x | x |
|  | C-peptide [nmol/L] |  |  | x |  | x | x |
|  | Insulin resistance from response to oral glucose tolerance test (oGTT) |  |  | x |  |  | x |
| **Blood lipids** | |  |  |  | | |  |
|  | Total cholesterol [mmol/l] |  |  | x |  | x | x |
|  | High-density lipoprotein cholesterol (HDL)^b^ [mmol/l] |  |  | x |  | x | x |
|  | Low density lipoprotein cholesterol (LDL) [mmol/l] |  |  | x |  | x | x |
|  | Triglycerides^b^ [mmol/l] |  |  | x |  | x | x |
| **Bone mass and architecture** | |  |  |  | | |  |
|  | Bone mineral content [g/cm and z-scores] for total body, lumbar spine, and femoral neck by DXA | x |  |  |  |  | x |
|  | Areal bone mineral density [g/cm^2^ and z-scores] for total body, lumbar spine, and femoral neck by DXA | x |  |  |  |  | x |
|  | Trabecular Bone Score (TBS) by DXA | x |  |  |  |  | x |
|  | Vertebral Fracture Assessment (VFA) by DXA | x |  |  |  |  | x |
|  | Total cross sectional bone area [mm^2^ and z-scores] of the distal and proximal sites of radius and tibia by pQCT | x |  |  |  |  | x |
|  | Cortical cross sectional bone area [mm^2^ and z-scores] of the proximal site of radius and tibia by pQCT | x |  |  |  |  | x |
|  | Total and trabecular volumetric bone mineral density [g/cm^3^ and z-scores] of the distal site of radius and tibia by pQCT | x |  |  |  |  | x |
|  | Cortical bone mineral density [g/cm^3^ and z-scores] of the proximal site of radius and tibia by pQCT | x |  |  |  |  | x |
| **Bone metabolism** | |  |  |  | | |  |
|  | C-terminal telopeptide of type I collagen (CTX) [ng/ml] |  |  | x |  | x | x |
|  | Serum osteocalcin [μg/l] |  |  | x |  | x | x |
|  | Uncarboxylated osteocalcin [μg/l] |  |  | x |  | x | x |
|  | Procollagen Type I N-Terminal Propeptide (PINP) [ng/ml] |  |  | x |  | x | x |
|  | Bone-specific alkaline phosphatase (BAP) [μg/l] |  |  | x |  | x | x |
| **Bone hormones** | |  |  |  | | |  |
|  | 25-Hydroxy-Vitamin D3 [ng/ml] |  |  | x |  | x | x |
|  | Intact parathyroid hormone (iPTH) [pg/ml] |  |  | x |  | x | x |
|  | Total Testosterone (TT) [nmol/l] |  |  | x |  | x | x |
|  | Estradiol (E2) [pmol/l] |  |  | x |  | x | x |
|  | Cortisol [nmol/l] |  |  | x |  | x | x |
|  | Free thyroxine (fT4) [pmol/l] |  |  | x |  | x | x |
|  | Thyroid-stimulating hormone (TSH) [mU/l] |  |  | x |  | x | x |
|  | Follicle-stimulating Hormone (FSH) [U/l] |  |  | x |  | x | x |
|  | Luteinizing hormone (LH) [U/l] |  |  | x |  | x | x |
|  | Insulin Like Growth Factor 1 (IGF-1) [nmol/l] |  |  | x |  | x | x |
|  | Insulin Like Growth Factor Binding Protein 3 (IGF-BP3) [nmol/l] |  |  | x |  | x | x |
| **Nutrition questionnaires** | |  |  |  | | |  |
|  | Vitamin D (supplements, sun exposure and nutrition) |  |  | x |  | x | x |
|  | Calcium intake [mg/day] |  |  | x |  | x | x |
|  | Protein intake [g/day] |  |  | x |  | x | x |
| **Aerobic fitness** (by cycle ergometer test) | |  |  |  | | |  |
|  | Peak oxygen uptake (VO2max)^b^ [ml/(kg*min) and % predicted] | x |  |  |  | x | x |
|  | Peak performance [Watt and % predicted] | x |  |  |  | x | x |
|  | Heart rate during exercise [beats per minute] | x |  |  |  | x | x |
|  | Blood pressure during exercise [mmHg] | x |  |  |  | x | x |
|  | Borg Rating of Perceived Exertion (RPE) during exercise [stage ranging from 1-10] | x |  |  |  | x | x |
|  | Respiratory exchange ratio during exercise [ratio] | x |  |  |  | x | x |
|  | Heart rate recovery [delta beats/min] | x |  |  |  | x | x |
| **Muscular strength** | |  |  |  | | |  |
|  | Hand grip strength in the left and right hand [kg] | x |  |  |  | x | x |
|  | Leg strength and endurance [repetitions/min] | x |  |  |  | x | x |
| **Pedometry^c^** | |  |  |  | | |  |
|  | Normal and aerobic steps per day [n steps/day] |  |  | ^c^ |  | ^c^ | ^c^ |
| **Accelerometry^c^** | |  |  |  | | |  |
|  | Total physical activity [counts/min] |  |  | ^c^ |  | ^c^ | ^c^ |
|  | Time spent in light, moderate and vigorous physical activities [minutes/day] |  |  | ^c^ |  | ^c^ | ^c^ |
|  | Sedentary behaviour [minutes/day] |  |  | ^c^ |  | ^c^ | ^c^ |
|  | Impact loading with ground reaction forces >2,3,4,5, and 6 G [n/day] |  |  | ^c^ |  | ^c^ | ^c^ |
| **Physical activity questionnaires** | |  |  |  | | |  |
|  | Motivation for physical activity |  |  | x |  | x | x |
|  | Physical activities of the previous 7 days |  |  | x | x | x | x |
|  | General physical activity questions |  |  | x |  |  |  |
| **Quality of life questionnaires** | |  |  |  | | |  |
|  | Health-related quality of life [T-score, range=0-100] |  |  | x |  | x | x |
|  | Fatigue and well-being [VAS] |  |  | x | x | x | x |
|  | Psychological distress [T-score, range=0-100] |  |  | x |  | x | x |
| **Personal History and current clinical status** | |  |  |  | | |  |
|  | Socio-demographic characteristics |  |  | x |  |  |  |
|  | Symptoms and late effects | x |  |  |  | x | x |
|  | Medications, treatments, hospitalizations and doctor visits [n and time to event in days] | x |  |  |  | x | x |
|  | Health behaviours |  |  | x |  |  | x |
|  | Vital parameters | x |  |  | x | x | x |
|  | Physical examination of lung, heart, abdomen, joints, legs, feet, ear, mouth, eyes, lymph nodes, neurological status | x |  |  |  | x | x |
|  | Maturation [Tanner stage] | x |  |  |  | x^d^ | x^d^ |
| **Adverse events** | |  |  |  | | |  |
|  | Adverse events and serious adverse events |  |  |  |  |  |  |
|  | Exercise related complications |  |  |  |  |  |  |
| **Intervention compliance^a^** (by online diary) | |  |  |  | | |  |
|  | Normal and aerobic steps per day [n steps/day] |  |  |  |  |  |  |
|  | Type, duration and intensity of exercises performed per day |  |  |  |  |  |  |
|  | Daily well-being and fatigue [VAS] |  |  |  |  |  |  |
|  | Hours of sleep per night [n/day] |  |  |  |  |  |  |

^a^ For participants of the intervention group only

^b^ Element of the cardiovascular disease risk composite score (primary outcome)

^c^ Assessed over 14 days between two study visits

^d^ Only if not adult at baseline (timepoint T0a)

*Abbreviations:* DXA, Dual energy x-ray absorptiometry; HDL, High Density Lipoprotein; Lab, Laboratory; LDL, Low Density Lipoprotein; mt, months; n.a., not applicable; Prot., Protein; pQCT, Peripheral quantitative computed tomography; USB, University Hospital Basel; VAS, Visual Analogue Scale; VO2peak, Peak Oxygen Uptake; Wpeak, Peak Watt Performance.
