# Supplementary material for: A partially supervised physical activity program for adult and adolescent survivors of childhood cancer (SURfit): study design of a randomized controlled trial [NCT02730767]
Source: BMC Cancer. 2017 Dec 5;17:822. doi: 10.1186/s12885-017-3801-8 (PMC5717834; doi:10.1186/s12885-017-3801-8)
Supplement: Supplementary file 3 — Measures and methods within SURfit at different time points. The table in Additional file 3 describes in detail the methods used for each measurement in SURfit. It is an extension of the (Additional file 2: Table S1) in the paper. (DOCX 81 kb) [file 12885_2017_3801_MOESM3_ESM.docx]

**Additional File 2: Measures and methods within SURfit at different time points**

| **Measure** | | **Time point (month)** | | | | **Method** |
| --- | --- | --- | --- | --- | --- | --- |
|  | | 0 | 3 | 6 | 12 |  |
| ***Cardiovascular disease risk*** | | | | | | |
| **Blood pressure / ECG** | |  | | | |  |
|  | Systolic & diastolic blood pressure^a^ [mmHg] | x | x | x | x | By automated oscillograph |
|  | 12-lead electrocardiogram (ECG) | x |  | x | x |  |
| **Anthropometry** | |  | | | |  |
|  | Height [cm] | x | x | x | x |  |
|  | Body mass [kg] | x | x | x | x |  |
|  | Body Mass Index [z-score] | x | x | x | x |  |
|  | Waist circumference^a^ [cm] | x | x | x | x |  |
|  | Absolute [kg] and relative [%] body lean and fat mass | x | x | x | x | Assessed by skinfold thickness with Calliper |
|  | Absolute [kg] and relative [%] body fat mass | x |  |  | x | Assessed by Dual X-Ray Absorptiometry (DXA) |
|  | Muscle cross-sectional area [cm^2^ and z-scores] at radius and tibia | x |  |  | x | Assessed by peripheral Quantitative Computed Tomography (pQCT) |
| **Glycaemic control** | | | | | |  |
|  | Fasting insulin [mIU/l] | x |  | x | x | Chemiluminescent enzyme immunoassay |
|  | Fasting glucose [mmol/l] | x |  | x | x | Hexokinase method (Modular) |
|  | Homeostasis Model Assessment Insulin Resistance (HOMA-IR)^a^ | x |  | x | x | Calculated from insulin [mIU/l] and glucose [mmol/l] |
|  | Glycated haemoglobin (HbA1C) [mmol/mol] | x |  | x | x | High efficiency fluid chromatography |
|  | C-peptide [nmol/L] | x |  | x | x | Fixed phase-chemical illuminesence-immuno-assay |
|  | Insulin resistance from response to oral glucose tolerance test (oGTT) | x |  |  | x | Assessment of fasting glucose [mmol/l] and fasting insulin [mIU/l] as well as blood glucose level [mmol/l] 2 hours after drinking of a glucose solution |
| **Blood lipids** | |  | | | |  |
|  | Total cholesterol [mmol/l] | x |  | x | x | Standard method on autoanalyzer (COBAS Integra 800) |
|  | High-density lipoprotein cholesterol (HDL)^a^ [mmol/l] | x |  | x | x |  |
|  | Low density lipoprotein cholesterol (LDL) [mmol/l] | x |  | x | x |  |
|  | Triglycerides^a^ [mmol/l] | x |  | x | x |  |
|  |  |  |  |  |  |  |
| ***Bone health*** | | | | | | |
| **Bone mass and architecture** | |  | | | |  |
|  | Bone mineral content [g/cm and z-scores] for total body, lumbar spine, and femoral neck | x |  |  | x | Assessed by DXA |
|  | Areal bone mineral density [g/cm^2^ and z-scores] for total body, lumbar spine, and femoral neck | x |  |  | x |  |
|  | Trabecular Bone Score (TBS) | x |  |  | x |  |
|  | Vertebral Fracture Assessment (VFA) | x |  |  | x |  |
|  | Total cross sectional bone area [mm^2^ and z-scores] of the distal and proximal sites of radius and tibia | x |  |  | x | Assessed by pQCT |
|  | Cortical cross sectional bone area [mm^2^ and z-scores] of the proximal site of radius and tibia | x |  |  | x |  |
|  | Total and trabecular volumetric bone mineral density [g/cm^3^ and z-scores] of the distal site of radius and tibia | x |  |  | x |  |
|  | Cortical bone mineral density [g/cm^3^ and z-scores] of the proximal site of radius and tibia | x |  |  | x |  |
| **Bone metabolism** | |  | | | |  |
|  | C-terminal telopeptide of type I collagen (CTX) [ng/ml] | x |  | x | x | Electro-chemiluminescence immunoassays (ECLIA) on the automated analyzer Elecsys |
|  | Serum osteocalcin [μg/l] | x |  | x | x | Electro-chemiluminescence immunoassays (ECLIA) on the automated analyzer Elecsys |
|  | Uncarboxylated osteocalcin [μg/l] | x |  | x | x |  |
|  | Procollagen Type I N-Terminal Propeptide (PINP) [ng/ml] | x |  | x | x |  |
|  | Bone-specific alkaline phosphatase (BAP) [μg/l] | x |  | x | x | Enzyme-immunoassay (EIA) on the IDS-iSYS (Immunodiagnostic Systems) |
| **Bone hormones** | |  | | | |  |
|  | 25-Hydroxy-Vitamin D3 [ng/ml] | x |  | x | x | Enzyme-immunoassay (EIA) on the IDS-iSYS (Immunodiagnostic Systems) |
|  | Intact parathyroid hormone (iPTH) [pg/ml] | x |  | x | x | Electro-chemiluminescence immunoassays (ECLIA) on the automated analyzer Elecsys |
|  | Total Testosterone (TT) [nmol/l] | x |  | x | x | Chemiluminescent enzyme immunoassays (ECLIA) on the automated analyzer COBAS e411 |
|  | Estradiol (E2) [pmol/l] | x |  | x | x |  |
|  | Cortisol [nmol/l] | x |  | x | x |  |
|  | Free thyroxine (fT4) [pmol/l] | x |  | x | x |  |
|  | Thyroid-stimulating hormone (TSH) [mU/l] | x |  | x | x |  |
|  | Follicle-stimulating Hormone (FSH) [U/l] | x |  | x | x |  |
|  | Luteinizing hormone (LH) [U/l] | x |  | x | x |  |
|  | Insulin Like Growth Factor 1 (IGF-1) [nmol/l] | x |  | x | x | Chemiluminescent enzyme immunoassays (ECLIA) on the IDS-iSYS |
|  | Insulin Like Growth Factor Binding Protein 3 (IGF-BP3) [nmol/l] | x |  | x | x |  |
| **Nutrition (questionnaire)** | |  | | | |  |
|  | Vitamin D (supplements, sun exposure and nutrition) | x |  | x | x | Standardized questions |
|  | Calcium intake [mg/day] | x |  | x | x | Standardized food frequency questionnaire |
|  | Protein intake [g/day] | x |  | x | x |  |
|  |  |  |  |  |  |  |
| ***Physical fitness*** | | | | | | |
| **Aerobic fitness** | |  | | | |  |
|  | Peak oxygen uptake (VO2max)^a^ [ml/(kg*min) and % predicted] | x |  | x | x | Cardiopulmonary exercise test (Godfrey protocol) on a cycle ergometer with metabolic cart |
|  | Peak performance [Watt and % predicted] | x |  | x | x |  |
|  | Heart rate during exercise [beats per minute] | x |  | x | x |  |
|  | Blood pressure during exercise [mmHg] | x |  | x | x |  |
|  | Borg Rating of Perceived Exertion (RPE) during exercise [stage ranging from 1-10] | x |  | x | x |  |
|  | Respiratory exchange ratio during exercise [ratio] | x |  | x | x |  |
|  | Heart rate recovery [delta beats/min] | x |  | x | x | Heart rate by ECG 1, 2, and 3 minutes post exercise |
| **Muscular strength** | |  | | | |  |
|  | Hand grip strength in the left and right hand [kg] | x |  | x | x | By hydraulic hand dynamometer |
|  | Leg strength and endurance [repetitions/min] | x |  | x | x | Assessed with the 1 minute sit-to-stand test |
|  |  |  |  |  |  |  |
| ***Physical activity*** | | | | | | |
| **Pedometry** | |  | | | |  |
|  | Normal and aerobic steps per day [n steps/day] | x |  | x | x | By pedometer |
| **Accelerometry** | |  | | | |  |
|  | Total physical activity [counts/min] | x |  | x | x | By accelerometer |
|  | Time spent in light, moderate and vigorous physical activities [minutes/day] | x |  | x | x |  |
|  | Sedentary behaviour [minutes/day] |  |  |  |  |  |
|  | Impact loading with ground reaction forces >2,3,4,5, and 6 G [n/day] | x |  | x | x |  |
| **Questionnaire** | |  | | | |  |
|  | Motivation for physical activity | x |  | x | x | Standardized self-reported questionnaire: Sport Motivation Inventory |
|  | Physical activities of the previous 7 days | x | x | x | x | Standardized self-reported questionnaire: 7-Day Physical Activity Recall Questionnaire (7D-PAR) |
|  | General physical activity questions | x |  |  |  | Standardized self-reported questions |
|  |  |  |  |  |  |  |
| ***Quality of life and mental health*** | | | | | | |
| **Questionnaire** | |  | | | |  |
|  | Health-related quality of life [T-score, range 0-100] | x |  | x | x | Standardized self-reported questionnaire: Short Form 36 (SF-36) |
|  | Fatigue and well-being | x | x | x | x | Standardized self-reported questionnaire: Checklist Individual Strength (CIS) and Visual Analogue Scale (VAS) |
|  | Psychological distress [T-score, range=0-100] | x |  | x | x | Standardized self-reported questionnaire: Brief Symptom Inventory (BSI-53) |
|  |  |  |  |  |  |  |
| ***Clinical status*** | | | | | | |
| **Personal History and Current clinical status** | |  | | | |  |
|  | Socio-demographic characteristics | x |  |  |  | Standardized self-reported questions |
|  | Symptoms and late effects | x |  | x | x | Standardized interview, medical record |
|  | Medications, treatments, hospitalizations and doctor visits [n and time to event in days] | x |  | x | x | Standardized interview, medical record |
|  | Health behaviours | x |  |  | x | Personal interview and standardized, self-reported questions |
|  | Vital parameters | x | x | x | x | Standardized physical examination |
|  | Physical examination of lung, heart, abdomen, joints, legs, feet, ear, mouth, eyes, lymph nodes, neurological status | x |  | x | x |  |
|  | Maturation [Tanner stage] | x |  | x^b^ | x^b^ |  |
|  |  |  |  |  |  |  |
| ***Safety outcomes*** | | | | | | |
| **Adverse events** | |  | | | |  |
|  | Adverse events and serious adverse events |  | x | x | x | Standardized interviews |
|  | Exercise related complications |  | x | x | x |  |

^a^ Element of the cardiovascular disease risk composite score

^b^ Only if not adult at baseline (T0)

*Abbreviations:* DXA, Dual energy x-ray absorptiometry; HDL, High Density Lipoprotein; Lab, Laboratory; LDL, Low Density Lipoprotein; n.a., not applicable; Prot., Protein; pQCT, Peripheral quantitative computed tomography; USB, University Hospital Basel; VO2peak, Peak Oxygen Uptake; Wpeak, Peak Watt Performance.
